# LAIR-1 Limits Neutrophilic Airway Inflammation

**DOI:** 10.3389/fimmu.2019.00842

**Published:** 2019-04-26

**Authors:** Kuldeep Kumawat, Ruben J. Geerdink, Marije P. Hennus, Mojtaba Abdul Roda, Ingrid van Ark, Thea Leusink-Muis, Gert Folkerts, Anita van Oort-Jansen, Alexandra Mazharian, Steve P. Watson, Frank E. Coenjaerts, Louis Bont, Linde Meyaard

**Affiliations:** ^1^Laboratory for Translational Immunology, Department of Immunology, University Medical Center Utrecht, Utrecht, Netherlands; ^2^Oncode Institute, University Medical Center Utrecht, Utrecht, Netherlands; ^3^Department of Pediatric Intensive Care, Wilhelmina Children's Hospital, University Medical Center Utrecht, Utrecht, Netherlands; ^4^Division of Pharmacology, Faculty of Science, Utrecht Institute for Pharmaceutical Sciences, Utrecht University, Utrecht, Netherlands; ^5^Centre for Cardiovascular Sciences, Institute for Biomedical Research, College of Medical and Dental Sciences, University of Birmingham, Birmingham, United Kingdom; ^6^Centre of Membrane Proteins and Receptors, Universities of Birmingham and Nottingham, Birmingham, United Kingdom; ^7^Department of Medical Microbiology, University Medical Center Utrecht, Utrecht, Netherlands; ^8^Department of Pediatrics, Wilhelmina Children's Hospital, University Medical Center Utrecht, Utrecht, Netherlands

**Keywords:** LAIR-1, neutrophils, RSV, airway, inflammation, bronchiolitis, cigarette smoke

## Abstract

Neutrophils are crucial to antimicrobial defense, but excessive neutrophilic inflammation induces immune pathology. The mechanisms by which neutrophils are regulated to prevent injury and preserve tissue homeostasis are not completely understood. We recently identified the collagen receptor leukocyte-associated immunoglobulin-like receptor (LAIR)-1 as a functional inhibitory receptor on airway-infiltrated neutrophils in viral bronchiolitis patients. In the current study, we sought to examine the role of LAIR-1 in regulating airway neutrophil responses *in vivo*. LAIR-1-deficient (*Lair1*^−/−^) and wild-type mice were infected with respiratory syncytial virus (RSV) or exposed to cigarette smoke as commonly accepted models of neutrophil-driven lung inflammation. Mice were monitored for cellular airway influx, weight loss, cytokine production, and viral loads. After RSV infection, *Lair1*^−/−^ mice show enhanced airway inflammation accompanied by increased neutrophil and lymphocyte recruitment to the airways, without effects on viral loads or cytokine production. LAIR-1-Fc administration in wild type mice, which blocks ligand induced LAIR-1 activation, augmented airway inflammation recapitulating the observations in *Lair1*^−/−^ mice. Likewise, in the smoke-exposure model, LAIR-1 deficiency enhanced neutrophil recruitment to the airways and worsened disease severity. Intranasal CXCL1–mediated neutrophil recruitment to the airways was enhanced in mice lacking LAIR-1, supporting an intrinsic function of LAIR-1 on neutrophils. In conclusion, the immune inhibitory receptor LAIR-1 suppresses neutrophil tissue migration and acts as a negative regulator of neutrophil-driven airway inflammation during lung diseases. Following our recent observations in humans, this study provides crucial *in-vivo* evidence that LAIR-1 is a promising target for pharmacological intervention in such pathologies.

## Introduction

The lungs are constantly exposed to potential pathogens and other harmful agents. To protect against sudden incursions, neutrophils patrol the lung capillaries. A rapid and robust neutrophil response is crucial to antimicrobial defense (1, 2). Neutrophilic inflammation is a common trait of some respiratory diseases. We have recently reviewed literature showing that due to the promiscuous cytotoxicity of neutrophils, excessive neutrophilic inflammation induces immune injury in viral infection (3). Therefore, balancing pathogen eradication with neutrophil-induced tissue injury is of the utmost importance to preserve tissue homeostasis. However, the mechanisms that regulate neutrophilic inflammation in the airways are still unclear.

Leukocyte-associated Ig-like receptor (LAIR)-1, also known as CD305, is an ITIM-bearing inhibitory receptor expressed on majority of immune cells (4). Mouse and human LAIR-1 share ~40% homology, potent inhibitory capacity and bind to collagen and collagen-like molecules (5–8). Circulating neutrophils do not express LAIR-1 on the cell surface, but surface expression can be induced by *in vitro* stimulation, suggesting that LAIR-1 is involved in the regulation of activated, tissue-infiltrated neutrophils (9). We recently identified the collagen receptor LAIR-1 as functional inhibitory receptor on airway neutrophils obtained from RSV bronchiolitis patients (10). Activated airway-infiltrated neutrophils, but not their resting circulating counterparts, express LAIR-1 at the cell surface. Resting neutrophils store LAIR-1 intracellularly in granules, which allows for rapid surface upregulation upon activation. Agonistic antibody-mediated ligation of LAIR-1 on patient airway neutrophils suppresses neutrophil extracellular traps (NET) formation *ex-vivo*. Ligands for LAIR-1 are abundant in the lungs, including collagen in the extracellular matrix and surfactant protein-D (SP-D, which contains a collagen-like domains, in the airway lumen (11, 12). We, therefore, hypothesized that LAIR-1 regulates neutrophilic inflammation in lung diseases to minimize tissue injury. However, patient studies do not allow for experimental settings required to investigate the *in-vivo* role of LAIR-1. To test this hypothesis, we used mouse models and examined two distinct lung diseases in which neutrophilic inflammation plays a key role, namely, acute viral bronchiolitis caused by RSV infection, and lung inflammation induced by short-term smoke-exposure.

## Materials and Methods

### Animals

*Lair1*^−/−^ mice were generated on the C57BL/6 background by Taconic Artemis as described (13). BALB/c mice were procured from Harlan (Horst, the Netherlands). All animal studies were approved by the Institutional Animal Care and Use Committee and carried out in accordance with the national and institutional guidelines.

### Mouse RSV Infection

Eight to Twelve-week-old female C57BL/6 *Lair1*^−/−^ mice or their wild-type littermates were intranasally infected with 1 × 10^7^ PFU of RSV-A2 in 50 μl PBS. RSV-A2 preparation, quantitative assay for RSV-A2 titration and RSV-A2 infection of mice, including, intranasal inoculation, termination, and sample collection, was performed as described previously (14, 15). Mice were sacrificed on day 2 or 5 post-infection.

### LAIR-Fc Administration

For LAIR-1 blocking experiments, recombinant mouse LAIR-1 fused with the Fc portion of mouse IgG2a was produced in-house. The Fc tail was mutated to prevent binding to Fc receptors and complement as described previously (16).

Eight to twelve-week-old female BALB/c wild-type mice were injected intraperitoneally with 200 or 400 μg of mouse LAIR-1-Fc chimeric protein in 100 μl PBS or PBS alone as control, 1 day before and 2 days after RSV infection. Intranasal RSV infection was performed as described above.

### Bronchoalveolar Lavage Collection and Processing

Bronchoalveolar lavage (BAL) fluid collection was performed by flushing the lungs 2 times with 1.0 ml of ice-cold PBS. BAL fluid was centrifuged; supernatants were stored at −80°C for further analyses. Total cell counts in BAL were determined using a Bürker-Türk hemocytometer. BAL cells were analyzed by flow cytometry or examined by light microscopy.

For differential cell analysis by light microscopy, at least 200 cells were counted to assign relative quantities of macrophages, lymphocytes, and neutrophils based on morphology after May–Grünwald–Giemsa stain.

RSV-A2 concentrations in BAL fluid supernatants were analyzed by real-time PCR as described previously (15). Mouse CXCL1 (KC) and IL-6 levels in BAL fluid supernatants were measured by ELISA (Peprotech, London, UK) according to the manufacturer's instructions.

### Cigarette-Smoke Exposure

Male, 8–12 weeks old, wild-type and *Lair1*^−/−^ C57BL/6 mice were randomly assigned to undergo cigarette smoke (CS) or control air exposure. Mice were exposed to whole body mainstream CS generated from standard research cigarettes (3R4F; 9.4 mg tar/0.726 mg nicotine, University of Kentucky) using a Watson-Marlow roller pump (323 E series, speed 35 RPM; Watson Marlow, Rotterdam, The Netherlands) that directed the CS into the exposure chamber (25 L of air volume). Carbon monoxide and oxygen levels in the exposure chamber were measured using a gas analyzer from Bacharach (PCA-3 series; Bacharach, New Kensington, PA, USA) carbon monoxide concentrations were held at 150–300 ppm and oxygen levels were kept at 20%. Mice were exposed to CS twice daily for a maximum of 30 min with a 5-h smoke-free interval, for 10 consecutive days. On the first day of CS exposure, mice were exposed to CS from 2 pairs of cigarettes, followed by CS of two times 3 cigarettes on the second day, CS of 4 and 5 cigarettes on the third day, CS of 5 and 6 cigarettes on the fourth day, and CS of two times 6 cigarettes on fifth and remaining days. Mice were weighed daily. On the final day, mice were sacrificed and BAL fluid was collected.

### Intranasal Instillation of CXCL1

Eight to Sixteen week-old male and female *Lair1*^−/−^ mice (on a C57BL/6 background) or their wild-type littermates were randomly assigned to be instilled with 0.1 μg or 0.5 μg CXCL1 (R&D Systems, Minneapolis, MN, USA) in 50 μl PBS or PBS alone. After 4 h, mice were sacrificed and BAL fluid was collected. Cells were counted and analyzed by flow cytometry as described below.

### Single-Cell Suspension Preparation

Spleen and lymph nodes were mechanically dissociated and filtered sequentially through 100 and 70 μm cell strainers (BD Biosciences, San Jose, CA, USA). Bone marrow was flushed from femurs and tibiae and filtered through 70 μm cell strainers. Lungs were mechanically dissociated using the gentleMACS Dissociator (Miltenyi Biotec, Leiden, the Netherlands) as recommended by the manufacturer, and were enzymatically digested for 30 min at 37°C with 0.13 Wünch U/ml of LiberaseTM (Roche, Basel, Switzerland) and 200 μg/ml DNAse I (Roche, Basel, Switzerland) in RPMI-1640. Subsequently, the digested lung cell suspension was passed through 70 μm cell strainers. Red blood cells were lysed by 10 min incubation at 4°C in ammonium chloride carbonate buffer containing 155 mM NH_4_Cl, 10 mM KHCO_3_, and 0.1 mM EDTA. Single-cell suspensions were analyzed by flow cytometry.

### Flow Cytometry

Single-cell suspensions from tissues and cells from BAL fluid were stained for surface markers for 30 min at 4°C in PBS containing 0.01% (m/v) sodium azide and 1% (m/v) BSA. Propidium iodide (0.3 μg/mL, Sigma, St. Louis, MO, USA) in PBS was used to distinguish vital cells. Samples were treated with rat anti-mouse CD16/CD32 antibody (clone 2.4G2, BD Biosciences, Basel, Switzerland) to block FcR-mediated non-specific antibody binding prior to incubation with fluorochrome-conjugated antibodies. Acquisitions were made with a BD Canto II or LSR Fortessa and analyzed using FlowJo software (version 10.0.7, Treestar).

### Statistics

The statistical significance of differences between two groups was calculated with the unpaired Mann-Whitney or Student's *t* test where appropriate and more than two groups were compared with the 2-way ANOVA or Kruskal-Wallis test as mentioned in the legends. Statistical analyses were performed using Prism 6 (GraphPad) software. *P-*values < 0.05 were considered significant and are marked in the graphs where applicable. All unmarked differences are non-significant.

## Results

### LAIR-1 Negatively Regulates Neutrophil Recruitment During RSV Infection

We recently demonstrated that LAIR-1 is a functional inhibitory receptor on airway-infiltrated neutrophils of RSV infection-induced bronchiolitis patients in an *ex-vivo* setting (10). We therefore hypothesized that LAIR-1 regulates the neutrophil response *in vivo* during viral bronchiolitis. To test this hypothesis, wild-type and *Lair1*^−/−^ C57BL/6 mice were intranasally inoculated with the RSV-A2 strain. Mice were sacrificed on day 2 or 5 post-infection and BAL was performed to assess the cellular airway infiltrate, cytokine levels, and viral load.

**Figure 1:**
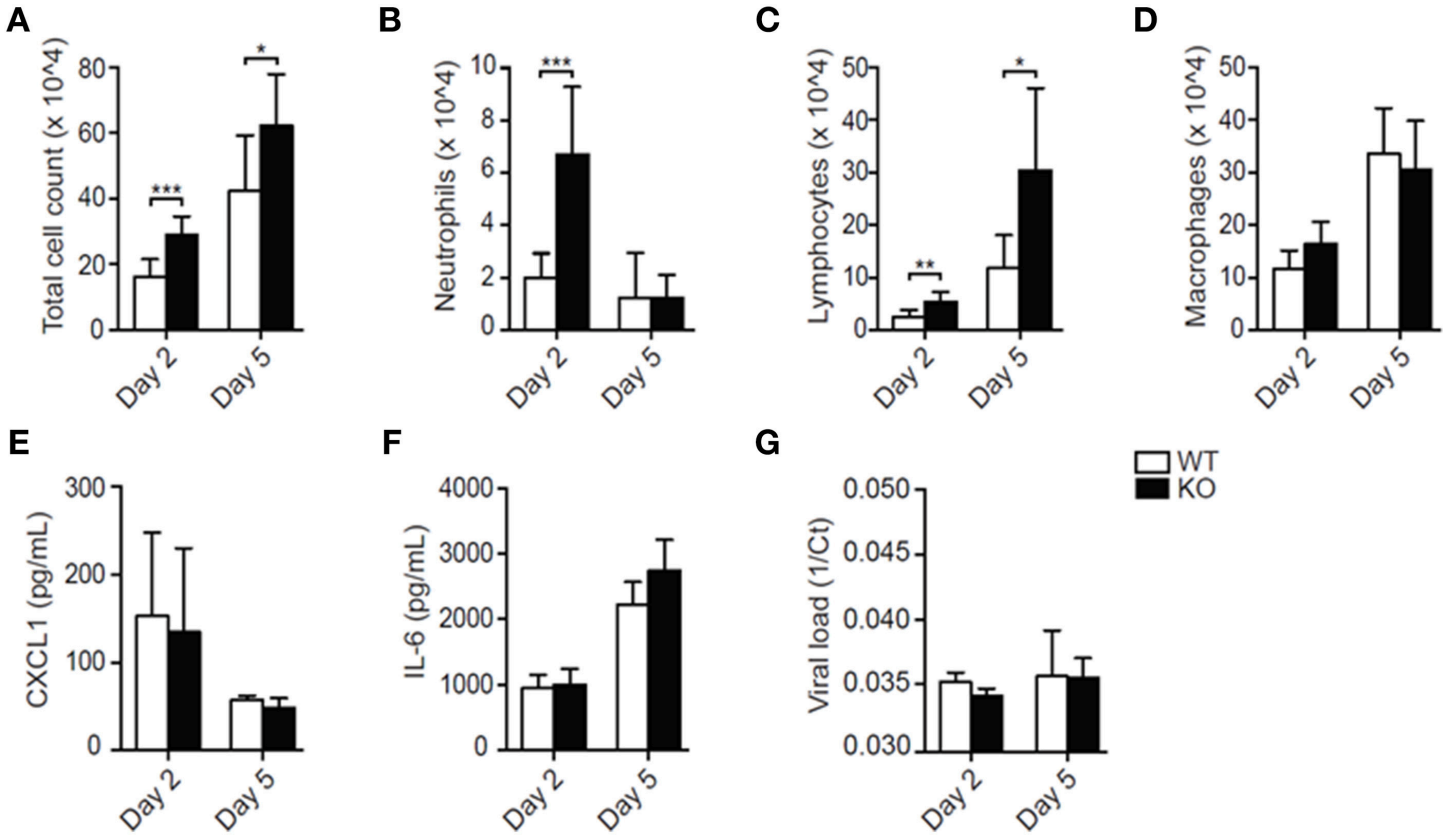
LAIR-1 regulates neutrophil and lymphocyte recruitment during RSV-A2 infection in mice. **(A–G)** Mice were inoculated with RSV-A2 and sacrificed on day 2 or 5. Total **(A)** and differential **(B–D)** BAL cell counts, BAL CXCL1 **(E)** and IL-6 **(F)** concentrations, and viral loads **(G)** were determined. Data are presented as means ± SD and represent 8 mice per group in 2 independent experiments. *p < 0.05, **p < 0.01, ***p < 0.001; unpaired Student's *t-test* with Welch's correction. WT, *wild-type* and KO, *Lair1*^−/−^ on C57BL/6 background.

Total leukocyte influx into the airways was notably increased in LAIR-1-deficient compared with wild-type mice at both the time points (Figure 1A). Early in infection, the increase in cell numbers was mostly contributed by enhanced neutrophil recruitment in LAIR-1-deficient mice, while at day 5 post-infection lymphocytes mainly constituted the infiltrating population (Figures 1B,C). There were no differences in macrophage recruitment between genotypes (Figure 1D). Thus, LAIR-1 limits neutrophil and lymphocyte recruitment during RSV infection *in vivo*.

Despite the enhanced leukocyte recruitment, concentrations of the major neutrophil chemoattractant CXCL1 and the inflammatory cytokine IL-6 were not increased in the BAL fluid of RSV-infected *Lair1*^−/−^ mice compared with wild-type mice (Figures 1E,F), nor were there differences in the viral load (Figure 1G). Thus, the data suggest that LAIR-1 negatively regulates neutrophil and lymphocyte recruitment during RSV infection without directly affecting the local inflammatory milieu or viral replication.

### Tissue-Infiltrated Neutrophils Express LAIR-1

**Figure 2:**
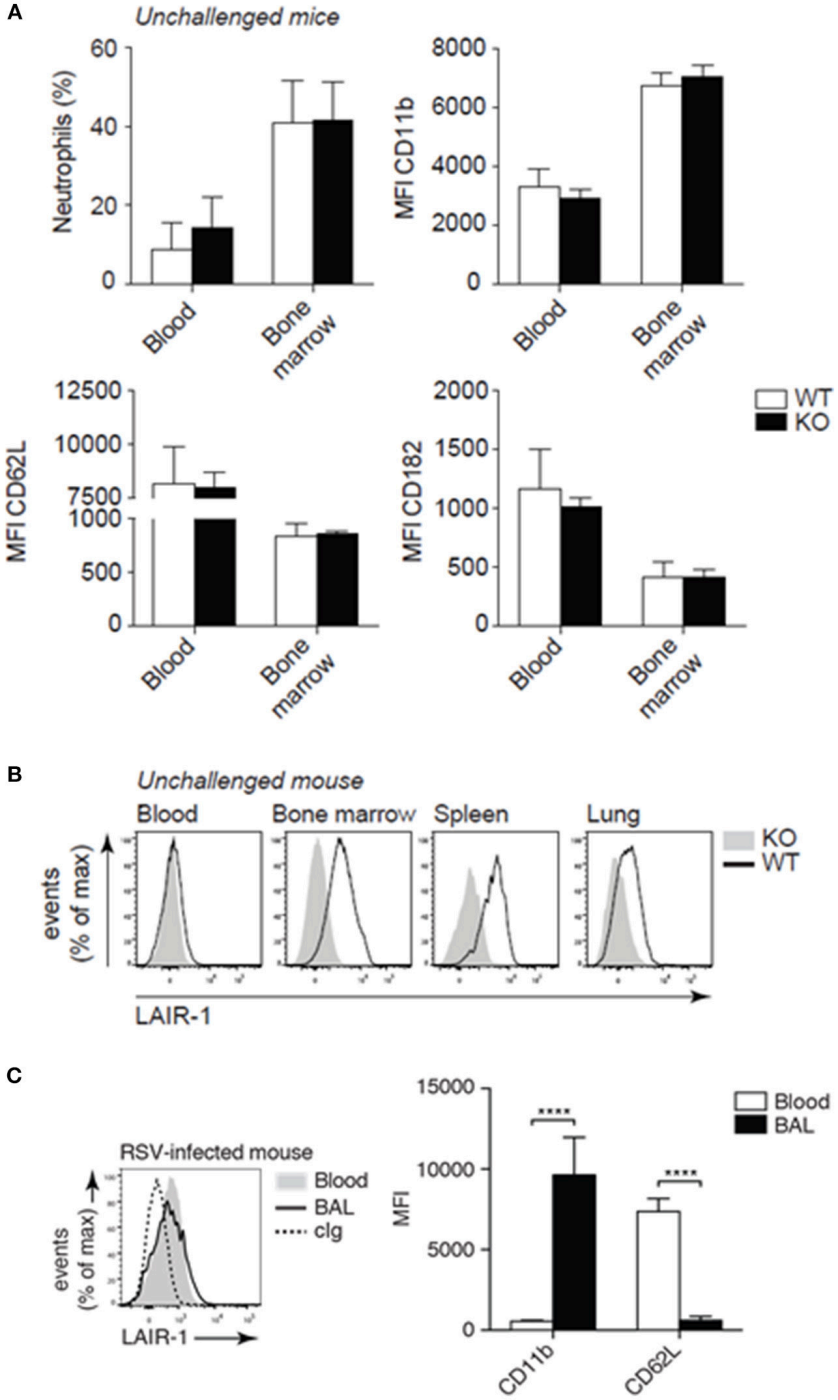
LAIR-1 expression and deficiency in mice. **(A–C)** Mouse leukocytes were examined for lineage and activation markers and LAIR-1 expression by flow cytometry. Neutrophils were identified based on characteristic forward- and side-light scatter properties and the expression of Ly-6G. **(A)** The percentage neutrophils (Ly-6G+) among total live leukocytes in blood and bone marrow as well as the expression of activation markers (CD11b, CD62L, and CD182) were compared by flow cytometry. **(B)** Flow cytometric analysis of LAIR-1 expression on blood and tissue (bone marrow, spleen, and lung) neutrophils. **(C)** Wild-type mice were inoculated with RSV and sacrificed 2 days post-infection. Expression of LAIR-1 and activation markers (CD11b and CD62L) was measured on BAL and blood neutrophils. ****p < 0.0001; 2-way ANOVA with Holm-Šídák multiple comparison correction. Data are representative of eight mice **(A, B)** or three independent experiments with at least three mice **(C)**. Error bars in **(A)** and **(C)** represent mean ± SD. WT, *wild-type* and KO, *Lair1*^−/−^ on C57BL/6 background; cIg, *isotype-matched control antibody*; MFI, *mean fluorescence intensity*.

To rule out basal differences, we performed immunophenotyping of unchallenged *Lair1*^−/−^ C57BL/6 mice and confirmed that there were little to no differences in the composition of immune cell populations (Supplemental Figures S1, S2) as described before (13, 17). Moreover, we demonstrate that unchallenged wild-type and *Lair1*^−/−^ mice did not differ in neutrophil numbers and activation state—indicated by the CD11b, CD62L, and CD182 markers—in either blood or bone marrow (Figure 2A). In unchallenged wild-type mice, circulating neutrophils did not express LAIR-1, whereas tissue-infiltrated neutrophils did (Figure 2B). However, upon infection with RSV, circulating neutrophils started to express LAIR-1 (Figure 2C). In line with the observation regarding tissue infiltrated neutrophils in unchallenged mice, airway-infiltrated neutrophils also expressed LAIR-1 and were highly activated—indicated by upregulation of CD11b and shedding of CD62L (Figure 2C). Thus, RSV infection induced LAIR-1 expression on circulating and airway-infiltrated neutrophils which may directly regulate the function/adhesion of neutrophils.

### Blocking LAIR-1-Ligand Interaction Enhanced Neutrophil Recruitment During RSV Infection

**Figure 3:**
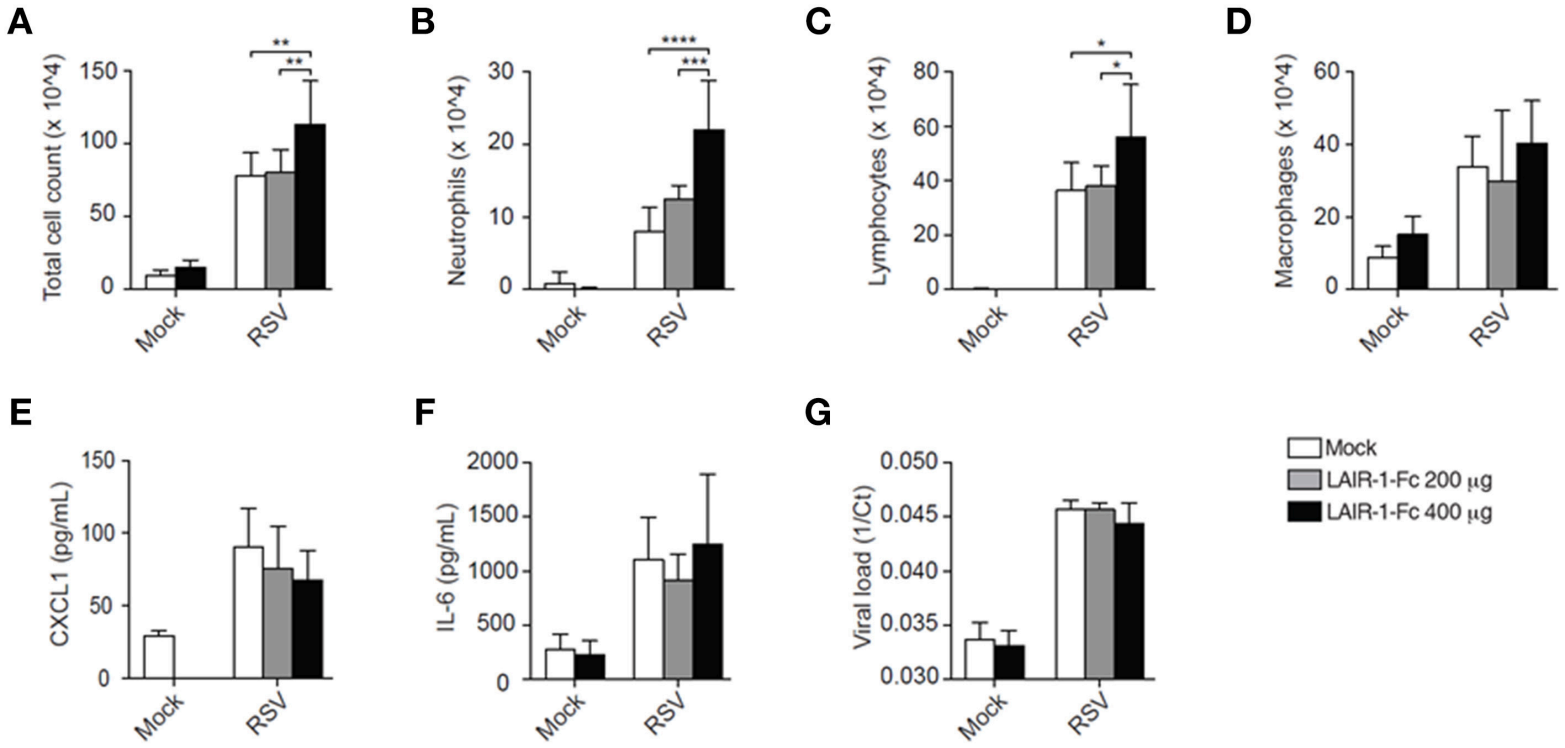
LAIR-1-Fc chimeric protein administration effects pulmonary neutrophil recruitment during RSV infection. **(A–G)** Wild-type BALB/c mice were inoculated intranasally with RSV and sacrificed on day 5. One day before RSV inoculation and 2 days after inoculation mice were treated intraperitoneally LAIR-1-Fc fusion protein or PBS (vehicle). Total **(A)** and differential **(B–D)** BAL cell counts, BAL CXCL1 **(F)** and IL-6 concentrations **(G)**, and viral load **(G)** were determined. Data are presented as means ± SD and represent 6 mice per group for panels **(A–G)** in 2 independent experiments. *p < 0.05, **p < 0.01, ***p < 0.001, ****p < 0.0001. 2-way ANOVA with Holm-Šídák multiple comparison correction.

To further rule out developmental differences in *Lair1*^−/−^ mice as cause of the observed phenotype, the interaction between endogenous LAIR-1 and its ligands was blocked during RSV infection by injecting wild-type mice with LAIR-1-Fc chimeric protein. Here, BALB/c mice rather than C57BL/6 mice were used as the former are more sensitive to RSV infection (18, 19). The results obtained with RSV-infected BALB/c mice in which endogenous LAIR-1-collagen interactions were blocked, mimic those of the RSV-infected *Lair1*^−/−^ C57BL/6 mice, when compared to their respective vehicle-treated or wild-type mice. The total cell count in the BAL of RSV-infected LAIR-1-Fc chimeric protein-treated mice was increased (Figure 3A). This was due to enhanced neutrophil and lymphocyte recruitment, whereas macrophage numbers remained unaffected (Figures 3B–D). Concentrations of CXCL1 and IL-6 as well as viral load were comparable between vehicle- and LAIR-1-Fc-treated mice (Figures 3E–G). The data further confirm that LAIR-1 negatively controls neutrophil and lymphocyte recruitment during RSV infection with no direct effect on the local inflammatory milieu or viral replication.

### LAIR-1 Limits Disease Severity and Neutrophil Airway Recruitment in Response to Cigarette-Smoke Exposure

**Figure 4:**
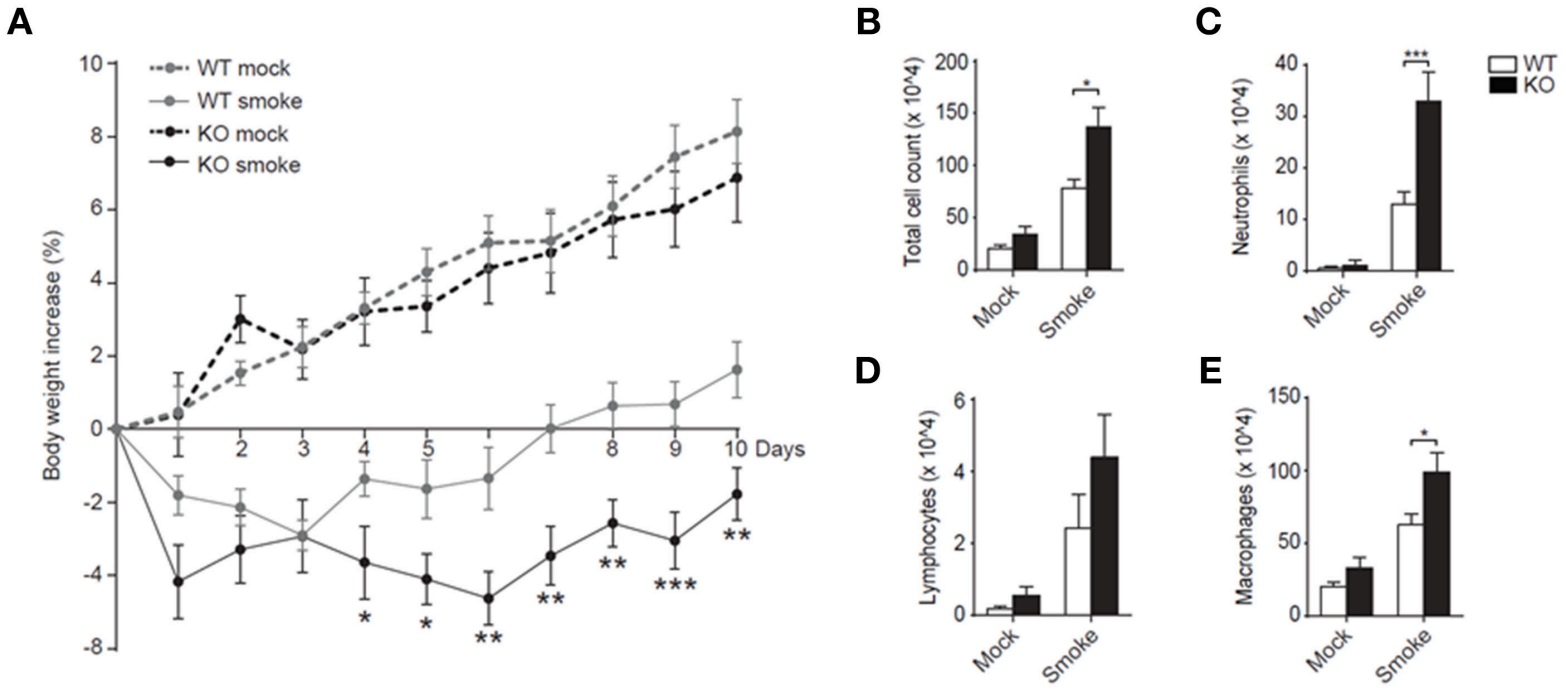
LAIR-1 regulates neutrophilic airway inflammation during cigarette-smoke exposure. **(A–E)** Mice were exposed to cigarette smoke or mock (air) in whole-body chambers twice daily for 10 consecutive days. **(A)** Body weight of mice was measured daily and percentage change relative to day 0 was calculated. Total **(B)** and differential counts **(C–E)** in BAL were determined after 10 days of cigarette-smoke exposure. Data are representative of at least 10 mice per group in 2 independent experiments. *p < 0.05, **p < 0.01, ***p < 0.001. The Mann-Whitney U test was used to calculate the statistical significance of differences between WT and KO mice **(A)** and a 2-way ANOVA with Holm-Šídák multiple comparison correction was used for the cell counts **(B,C)**. WT, *wild-type* and KO, *Lair1*^−/−^ on C57BL/6 background.

Despite the enhanced neutrophil recruitment during RSV infection in mice that lack LAIR-1 signaling, there was no effect on disease severity as measured by weight loss (data not shown). However, RSV pathophysiology in humans and mice is notably different. While neutrophils dominate the cellular inflammatory response in RSV bronchiolitis patients, with neutrophils comprising ≥80% of infiltrating leukocytes (20–22), lymphocytes are more prominent in the airways of mice during experimental RSV infection (Figures 1A–D, 3A–D). We hypothesized that in a genuine neutrophil-driven disease model the loss of LAIR-1-mediated immune regulation and the corresponding increase in neutrophil infiltration would exacerbate disease severity. Therefore, we employed another model of neutrophilic airway inflammation and exposed wild-type and *Lair1*^−/−^ C57BL/6 mice to cigarette smoke. Smoke-exposed *Lair1*^−/−^ mice lost more body weight and showed delayed and significantly attenuated recovery as compared to their wild-type counterparts (Figure 4A). In line with this, BAL fluid analysis revealed that *Lair1*^−/−^ mice had significantly higher lung immune infiltration in response to smoke-exposure as compared to the wild-type (Figure 4B). This increase in total cell influx was contributed by increased recruitment of neutrophils, macrophages and lymphocytes to *Lair1*^−/−^ lungs (Figures 4C–E). The increase in BAL cell counts in *Lair1*^−/−^ mice was highest for neutrophils (~3 fold) followed by macrophages (~2 fold), while the increase in lymphocytes was modest and failed to reach statistical significance. We confirmed that airway-infiltrated neutrophils of smoke-exposed mice expressed LAIR-1 and were highly activated (Supplemental Figure S2). Thus, LAIR-1 limits neutrophil recruitment to the airways during cigarette smoke-induced lung inflammation and regulates disease severity.

### LAIR-1 Directly Suppresses Neutrophil Migration

**Figure 5:**
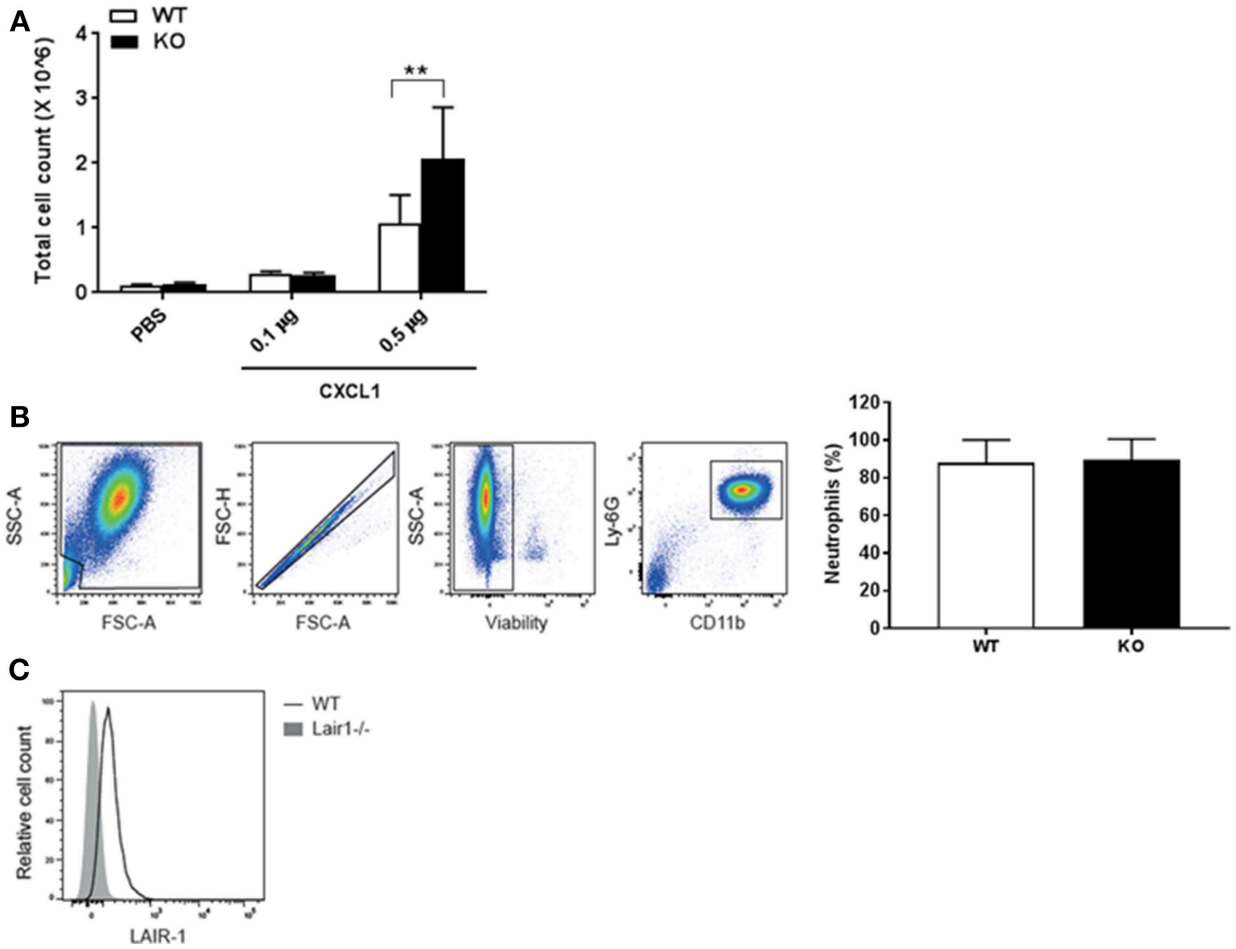
LAIR-1 directly controls neutrophil migration. **(A–C)** Mice were intranasally instilled with CXCL1 or vehicle (PBS). After 4 h mice were sacrificed and BAL was performed. **(A)** Total cell counts in BAL fluid were determined and represented as mean ± SD (*n* = 4–8 mice per group per genotype; 2 independent experiments). Differences between WT and KO in cell influx at CXCL1 (0.5 μg) were consistent in both experiments. **p < 0.01; 2-way ANOVA with Tukey's multiple comparison test. **(B,C)** Cells in BAL fluid were analyzed by flow cytometry. **(B)** Neutrophils were identified by concurrent Ly-6G and CD11b expression; graph represents neutrophils as percent of total live leukocytes in BAL fluid after 0.5 μg CXCL1 administration in WT and KO mice, mean ± SD and, **(C)** a representative histogram of LAIR-1 expression on BAL fluid neutrophils. WT, *wild-type* and KO, *Lair1*^−/−^ on C57BL/6 background.

The local inflammatory milieu in the lung, as reflected by CXCL1 and IL-6 production, was not directly regulated by LAIR-1 (Figures 1E,F, 3E,F). Since the lung is enriched in LAIR-1 ligands such as collagen, SP-D and airway-infiltrated neutrophils in both the RSV infection and the smoke exposure model express LAIR-1 (Figure 2C; Supplemental Figure S3), we hypothesized that LAIR-1 can directly limit the migratory capacity of neutrophils. To examine this hypothesis, the lungs of wild-type and *Lair1*^−/−^ C57BL/6 mice were instilled with the neutrophil chemoattractant CXCL1 by intranasal administration and analyzed for the BAL fluid cellular infiltrates. We observed significantly higher total BAL cell influx in *Lair1*^−/−^ mice as compared to wild-type mice in response to 0.5 μg of CXCL1 (Figure 5A). Flow cytometry confirmed that CXCL1 specifically attracted neutrophils, as they constitute ~88% of total cell population in BAL fluid (Figure 5B), which were highly activated and expressed LAIR-1 (in wild-type mice) (Figure 5C). These data demonstrate that LAIR-1 intrinsically limits neutrophil infiltration of the airways, thereby controlling neutrophilic airway inflammation.

## Discussion

The pulmonary immune response must protect against the ever-present threat of pathogens, while limiting immune-induced tissue damage to allow for gas exchange. Neutrophils are crucial for antimicrobial defense but such responses must be tightly regulated to prevent bystander damage. The mechanisms underlying the regulation of neutrophil activation and responses are incompletely understood. We have recently identified LAIR-1 as an inhibitory receptor on activated airway neutrophils which limits NET formation during RSV bronchiolitis (10). In the current study, we investigated the role of LAIR-1 in regulating airway inflammation using two different models of neutrophil pre-dominant lung diseases. Using a mouse model of RSV bronchiolitis, we demonstrate that LAIR-1 functions as a negative regulator of airway inflammation as LAIR-1 deficiency or administration of Lair1-Fc chimeric protein led to enhanced recruitment of neutrophils and lymphocytes. Similar results were obtained in the cigarette smoke exposure model where LAIR-1 deficient mice show marked increased neutrophilia. Our study, thus, underlines a key regulatory role of LAIR-1 in limiting neutrophilic inflammation in lung diseases.

Ligands for LAIR-1 are abundant in the lungs. En route to the airways, for instance in response to RSV infection or irritants in smoke, neutrophils will traverse the extracellular matrix, which contains collagen. In the airway lumen, neutrophils will encounter pulmonary surfactant-associated protein-D (SP-D) and C1q, which possess a collagen-like domain (11, 12). The interaction of LAIR-1 with the ligands present in the extracellular matrix may limit the recruitment of neutrophils to the airways. In support hereof, we observed an increased neutrophil recruitment to airways of RSV-infected and smoke-exposed mice that lack functional LAIR-1. Interestingly, this enhanced neutrophilia in *Lair1*^−/−^ mice was not associated with augmented chemokine production—for example, by LAIR-1-expressing alveolar macrophages—that attracts more neutrophils. There were no changes in the abundance of CXCL1, a potent neutrophil chemoattractant (23) and IL-6, a major pro-inflammatory cytokine, among wild-type and *Lair1*^−/−^ mice. This is suggestive for an enhanced intrinsic cellular migratory capacity in the absence of an inhibitory interaction of LAIR-1 with extracellular matrix. Indeed, in response to an intrapulmonary challenge with the neutrophil chemoattractant CXCL1 (23), neutrophil recruitment was strongly enhanced in *Lair1*^−/−^ mice compared with wild-type mice, thereby demonstrating a neutrophil-intrinsic role for LAIR-1 in migration to the airways. Thus, during an active inflammation and in response to chemoattractant stimuli, LAIR-1 interaction with its ligands impedes neutrophil migration in wild-type mice but not in *Lair1*^−/−^ mice.

A prior study of *Lair1*^−/−^ mice did not reveal an overt clinical phenotype in multiple lymphocyte-driven disease models (13, 17). Similar to our study (Supplemental Figure S3), LAIR-1 deficiency had little effect on the composition of immune cell populations or neutrophil activation state in unchallenged mice. However, in the previous studies neutrophils were not extensively studied. In contrast, we examined two different models of lung diseases where neutrophils are a dominant contributor, namely RSV bronchiolitis and smoke-induced inflammation. In both cases, LAIR-1 regulated neutrophilic lung inflammation. We observed no differences in disease severity between RSV-infected *Lair1*^−/−^ and wild-type mice. A possible explanation for this is that while in RSV bronchiolitis patients, the immune response to RSV is characterized by massive infiltration of neutrophils into the airways—≥80% of infiltrating leukocytes are neutrophils (20–22)—the contribution of airway infiltrating neutrophils on disease severity is notably less pronounced in mice (24). However, in response to cigarette smoke exposure, a bona fide neutrophil-driven lung inflammation model in mice, the enhanced neutrophilic inflammation in LAIR-1-deficient mice was accompanied by worsened weight loss and retarded recovery underlining the critical role of LAIR-1.

Neutrophils are crucial to antimicrobial defense, but cytotoxic effector mechanisms such as protease secretion, reactive oxygen species production, and NET formation cause bystander tissue damage (1–3, 25). Therefore, excessive neutrophilic inflammation is harmful. Major lung diseases, including RSV bronchiolitis and chronic obstructive pulmonary disease (COPD), are characterized by a massive neutrophilic inflammation (26). However, the regulatory mechanisms hereof are not yet fully elucidated. A better understanding of how neutrophilic inflammation is regulated could reveal potential targets for pharmaceutical intervention.

In human neutrophils, LAIR-1 is stored in intracellular granules and is rapidly recruited to the surface upon activation (10), presenting a plausible mechanism that would ensure a proper balance between neutrophil function and tissue injury. Under steady state, a low or lack of expression of LAIR-1 would ensure neutrophil activation against invading pathogens whereas rapid recruitment of LAIR-1 from intracellular stores would promptly impede the neutrophil influx and limit the tissue injury during neutrophilic inflammation. Whereas, our study shows that LAIR-1, indeed, impedes neutrophil influx during active inflammation in mouse models, the mechanisms of LAIR-1 surface expression on mouse neutrophils remains to be investigated.

Our study sheds light on a novel regulatory mechanism involved in neutrophilic inflammation but has several limitations. First, we cannot rule out the contribution of other LAIR-1-expressing immune cell populations, such as lymphocytes and macrophages, to the observed phenotypes. In addition to increased neutrophil infiltration, we also see increased recruitment of lymphocytes after RSV infection and increased number of macrophages after smoke-exposure in the airways of LAIR-1-deficient mice. Possibly, the augmented neutrophil response directly contributes to the subsequent increase in lymphocyte and macrophage influx. Indeed, neutrophils, by depositing CXCL12-containing vesicular trails during migration, are critical to the recruitment of T cells to the airways of influenza virus-infected mice (27). Whereas, both lymphocytes and macrophages could possibly contribute to increased disease severity, increased airway infiltration of neutrophils remains a consistent observation in LAIR-1-deficient mice in both RSV and smoke-exposure models.

Second, we measured a limited number of cytokines. Also, we did not investigate a direct effect of LAIR-1 on mouse neutrophil functions such as NET formation and migration *in-vitro*. Which aspect of the observed increased airway infiltration of neutrophils aggravates disease severity, remains unresolved. These questions require further investigation. The strength of our study lies in being the first to discern the role of LAIR-1 in neutrophil-predominant airway diseases *in vivo*. We show that LAIR-1 acts as a crucial regulator of neutrophils and is therefore a potential target for pharmacological intervention in neutrophil-driven lung diseases.

## Ethics Statement

The study was carried out in accordance with the national and international guidelines. The protocols were approved by the Institutional Animal Care and Use Committee of Utrecht University/University Medical Center Utrecht, the Netherlands.

## Author Contributions

KK and RG acquired, analyzed and interpreted data and wrote the manuscript. MH, MR, AvO-J, IvA, and TL-M performed mouse experiments. GF, AM, SW, and FC provided essential tools and technical insights. LB and LM designed the study, interpreted the data, and edited the manuscript. All authors reviewed and approved the manuscript.

### Conflict of Interest Statement

LB and LM have regular interaction with pharmaceutical and other industrial partners. They have not received personal fees or other personal benefits. LB's institute has received major funding (>€100,000 per industrial partner) for investigator-initiated studies from AbbVie, MedImmune, Janssen, the Bill and Melinda Gates Foundation and MeMed Diagnostics. LB's institute has received minor funding participation in trials by Regeneron and Janssen since 2015 (total annual estimate <€20,000). LB received minor funding for consultation and invited lectures by AbbVie, MedImmune, Ablynx, Bavaria Nordic, MabXience, Novavax, Janssen (total annual estimate <€20,000). LM's institute has received funding for investigator-initiated studies from Nextcure, Boehringer Ingelheim, Ono Pharmaceuticals, Ablynx and Janssen. LM received minor funding for consultation from Novo Nordisk, Biogen and Boehringer Ingelheim. The remaining authors declare that the research was conducted in the absence of any commercial or financial relationships that could be construed as a potential conflict of interest.
