# Supplementary material for: LAIR-1 Limits Neutrophilic Airway Inflammation
Source: Front Immunol. 2019 Apr 26;10:842. doi: 10.3389/fimmu.2019.00842 (PMC6497752; doi:10.3389/fimmu.2019.00842)
Supplement: Supplementary file 1 [file Data_Sheet_1.PDF]

Supplemental Figure S1

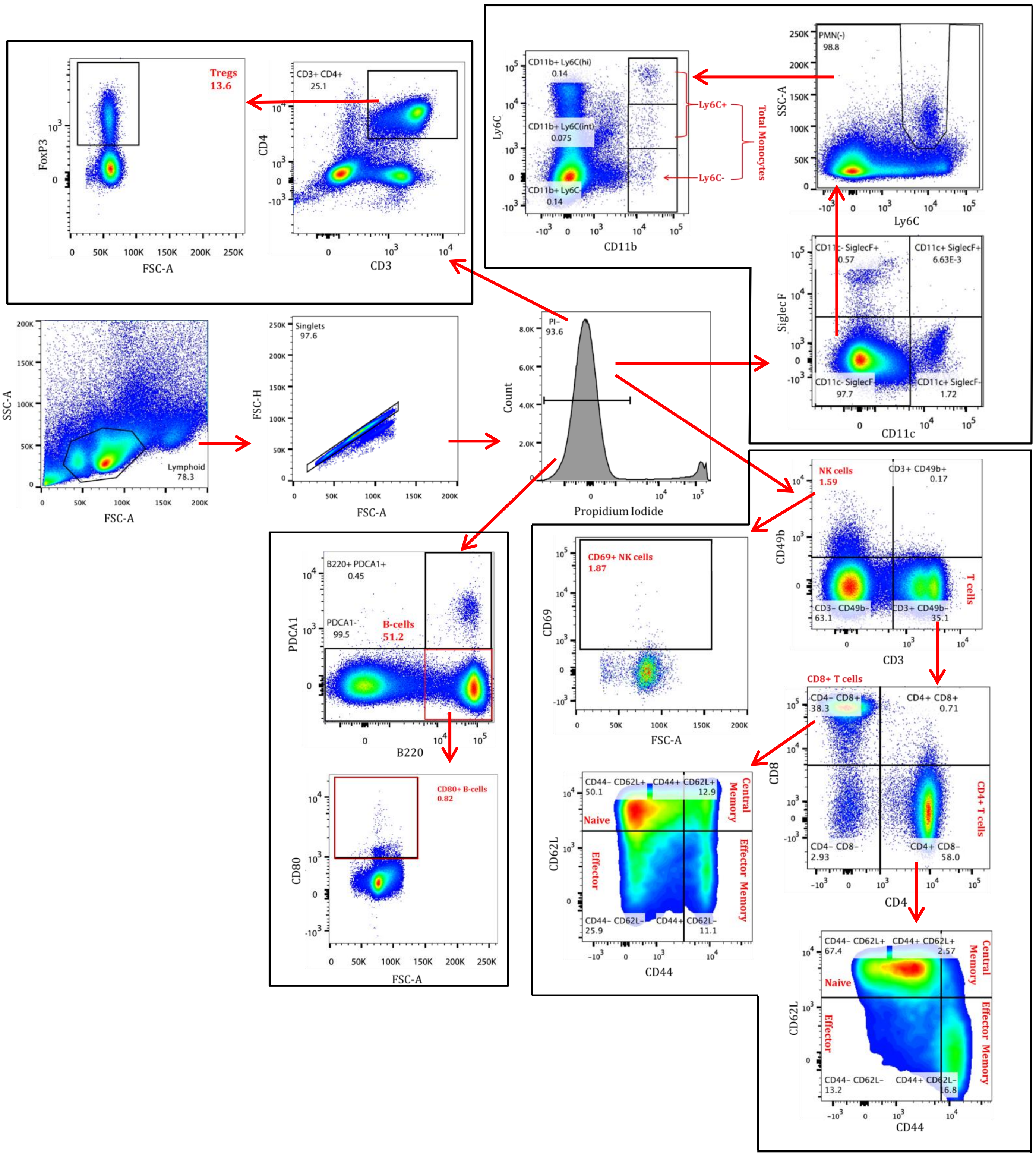

Supplemental Figure S1. Representative FACS gating strategy for immunophenotyping shown in Supplemental Figure S2.

Supplemental Figure S2

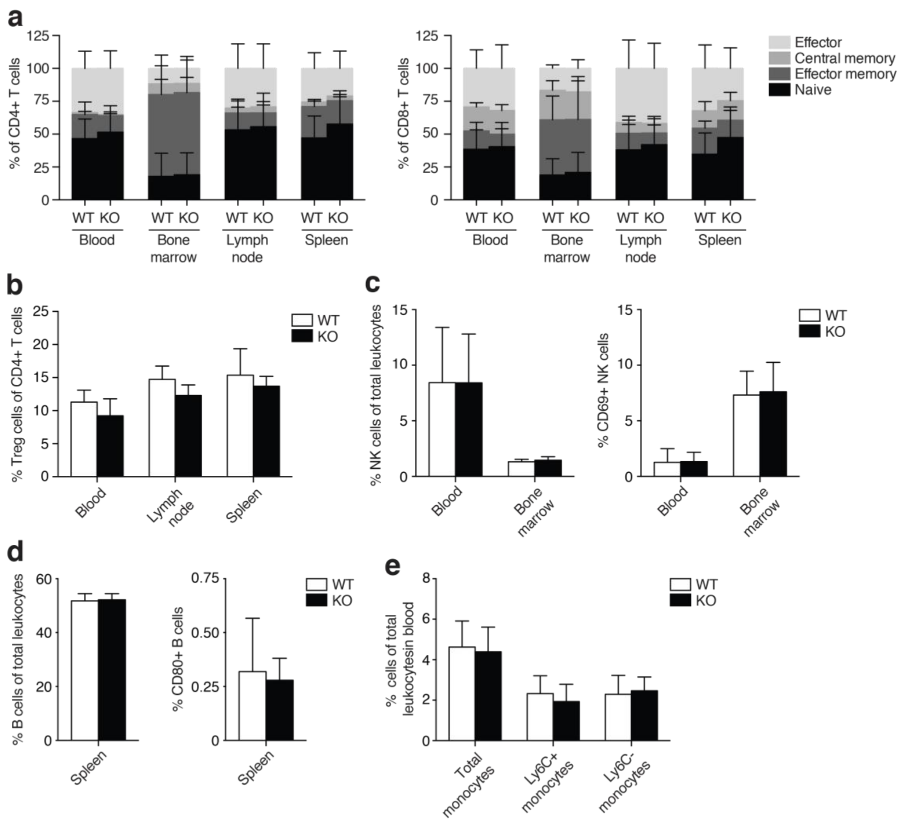

Supplemental Figure S2. No immunological differences between unchallenged mice. (a-e) Blood and tissue leukocytes of unchallenged WT and KO mice were stained for lineage and activation markers and analyzed by flow cytometry. (a) The percentages of naive (CD44$^-$ CD62L$^+$), effector memory (CD44$^+$ CD62L$^-$), central memory (CD44$^+$ CD62L$^+$), and effector (CD44$^-$ CD62L$^-$) cells among CD4$^+$ and CD8$^+$ T cell (CD3$^+$) populations were determined in blood, bone marrow, lymph nodes, and spleen. (b) The percentage of T_{reg} cells in the CD4$^+$ T cell population of blood, lymph nodes, and spleen of unchallenged WT and KO mice were determined by intracellular staining of FoxP3. (c) The percentage of CD49b$^+$ natural killer (NK) cells present among total leukocytes and the expression of activation marker CD69 on NK cells in blood and bone marrow. (d) The percentage of B cells present among total leukocytes from spleen and the percentage of splenic B cells that express the activation marker CD80. (e) The percentages of total, classical (Ly-6C$^+$), and non-classical (Ly-6C$^-$) monocytes present among blood leukocytes. WT, *wild-type* and KO, *Lair1*$^{-/-}$ on C57BL/6 background.

__**Supplemental Figure S3**__

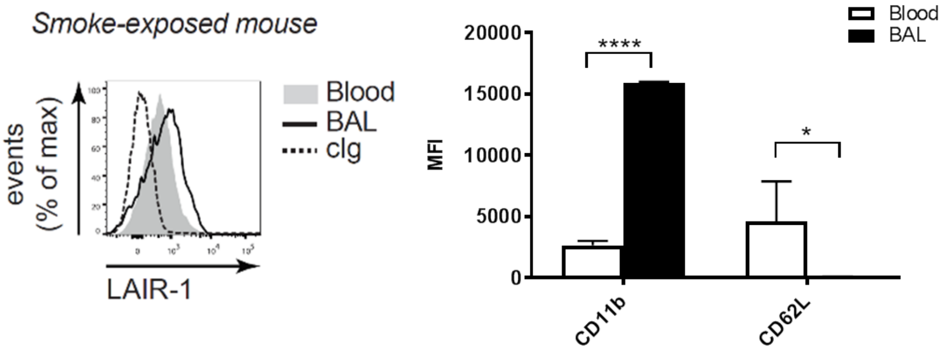

Supplemental Figure S3. Repeated cigarette smoke exposure induces LAIR-1 expression on mouse neutrophils. Wild-type C57BL/6 mice were repeatedly exposed to cigarette smoke during 10 days. Expression of LAIR-1 and activation markers (CD11b and CD62L) was measured on BAL and blood neutrophils by flow cytometry. Graph represents mean ± SD. * $p<0.01$ and **** $p<0.001$; 2-way ANOVA with Holm-Šídák multiple comparison correction. cIg, *isotype-matched control antibody*; MFI, *mean fluorescence intensity*.
